# Sir W. R. Gowers on Syphilitic Diseases of the Nervous System

**Published:** 1902-11-29

**Authors:** 


### SIR W. R. GOWERS ON SYPHILITIC DISEASES OF THE NERVOUS SYSTEM.

In a lecture recently delivered at the London Polyclinic, Sir William Gowers insisted afresh upon the fact that we know nothing of true syphilitic processes affecting the nerve elements themselves and curable by the customary methods. It is the supporting neuroglia, the membranes which blend with it, and the walls of the vessels, which form the seat of essential syphilitic changes in the nervous system, not the nerve elements themselves. Such changes, however, as take place in these connective tissue and vascular structures are never the direct cause of the symptoms. Nervous symptoms are always due to secondary consequences in the nerve elements resulting from interference with their function or nutrition by the morbid changes in the tissues by which they are surrounded or in the vessels by which they are fed, and these consequences are simple in nature and such as any other disease in the same situation might cause. Thus when a gumma presses on the spinal cord, it causes paraplegia just in the same way as would an equivalent pressure from any other form of tumour, and when syphilitic arterial disease narrows a blood-vessel, the lumen of which ultimately becomes closed by a simple clot, the resulting softening, by which symptoms are produced, is a simple softening such as would be produced by arterial obstruction arising from any other cause. Syphilis, however, is not without influence even upon the nerve elements themselves. They may suffer as a result of syphilis, but only by way of a slow degeneration, which is probably due to a post-syphilitic toxin, and is not only not benefited, but is sometimes accelerated or even induced by energetic specific treatment. It must not be forgotten that in some cases symptoms of this post-syphilitic degeneration may occur even during the period in which true syphilitic processes are going on; in other words, that the two stages or processes may overlap. For such cases treatment is, of course, necessary.

Recognising, then, that so far as the specific action of syphilis is concerned it affects the connective and vascular structures, and that the nervous symptoms are the result of the injury so caused to the associated nervous tissue, injury which may last long after its cause has ceased to act, it is evident that the persistence of symptoms by no means indicates persistence of the essential syphilitic processes of which the symptoms are the secondary consequences, and by no means necessarily points to perseverance in anti-syphilitic treatment.

As to the nature of such treatment, modern discovery has not added to that by mercury and iodide of potassium. That treatment should be energetic and brief. No other method is so good as that by inunction, but it is important to remember that the amount of mercury taken is but little guide to the amount retained in the tissues, owing to the extent to which elimination varies in different persons, and that unless mercury is pushed so as to produce a physiological effect it is impossible to be sure that enough is retained in the blood to influence the tissues. Hence the inunction should be carried to such an extent that in the course of 10 days a very slight affection of the gums should be obtained, and this should be kept up for three weeks. After this potassium iodide may be given. When there is need for energetic action iodide may be given for a few days at the beginning of the course, but as a rule it should not be given simultaneously with mercury, as it increases elimination. But neither mercury nor iodide should be continued for more than six or eight weeks. It is better that a course of iodide should be repeated for two or three weeks every four or six months for some years, whether the patient has symptoms or not, than to continue its administration over a long period.

